# Establishment and characterization of patient-derived xenografts as paraclinical models for head and neck cancer

**DOI:** 10.1186/s12885-020-06786-5

**Published:** 2020-04-15

**Authors:** Han Na Kang, Jae-Hwan Kim, A-Young Park, Jae Woo Choi, Sun Min Lim, Jinna Kim, Eun Joo Shin, Min Hee Hong, Kyoung-Ho Pyo, Mi Ran Yun, Dong Hwi Kim, Hanna Lee, Sun Och Yoon, Da Hee Kim, Young Min Park, Hyung Kwon Byeon, Inkyung Jung, Soonmyung Paik, Yoon Woo Koh, Byoung Chul Cho, Hye Ryun Kim

**Affiliations:** 1 JE-UK Institute for Cancer Research, JEUK Co. Ltd., Gumi-City, Kyungbuk South Korea; 2 Severance Biomedical Science Institute, Yonsei University College of Medicine, Seoul, South Korea; 3 Division of Medical Oncology, Department of Internal Medicine, CHA Bundang Medical Center, Seongnam-si, South Korea; 4 Department of Pathology, Yonsei University College of Medicine, Seoul, South Korea; 5 Yonsei Cancer Center, Division of Medical Oncology, Yonsei University College of Medicine, 50 Yonsei-ro, Seodaemun-gu, Seoul, 120-752 South Korea; 6 Department of Radiology, Severance Hospital, Yonsei University College of Medicine, Seoul, South Korea; 7 Department of Otorhinolaryngology, Yonsei University College of Medicine, 50 Yonsei-ro, Seodaemun-gu, Seoul, 120-752 South Korea; 8 Department of Otolaryngology-Head and Neck Surgery Korea, University College of Medicine, Seoul, South Korea; 9 Department of Biostatistics and Medical Informatics, Yonsei University College of Medicine, Seoul, South Korea

**Keywords:** Patient-derived xenograft, Biomarker, Head and neck cancer, Squamous cell cancer

## Abstract

**Background:**

We investigated whether head and neck squamous cell carcinoma (HNSCC) patient-derived xenografts (PDXs) reaffirm patient responses to anti-cancer therapeutics.

**Methods:**

Tumors from HNSCC patients were transplanted into immunodeficient mice and propagated via subsequent implantation. We evaluated established PDXs by histology, genomic profiling, and in vivo anti-cancer efficacy testing to confirm them as the authentic in vivo platform.

**Results:**

From 62 HNSCCs, 15 (24%) PDXs were established. The primary cancer types were tongue (8), oropharynx (3), hypopharynx (1), ethmoid sinus cancer (1), supraglottic cancer (1), and parotid gland (1); six PDXs (40%) were established from biopsy specimens from advanced HNSCC. PDXs mostly retained donor characteristics and remained stable across passages. *PIK3CA* (H1047R), *HRAS* (G12D), and *TP53* mutations (H193R, I195T, R248W, R273H, E298X) and *EGFR*, *CCND1*, *MYC*, and *PIK3CA* amplifications were identified. Using the acquisition method, biopsy showed a significantly higher engraftment rate when compared with that of surgical resection (100% [6/6] vs. 16.1% [9/56], *P* < 0.001). Specimens obtained from metastatic sites showed a significantly higher engraftment rate than did those from primary sites (100% [9/9] vs. 11.3% [6/53], *P* < 0.001). Three PDX models from HPV-positive tumors were established, as compared to 12 from HPV-negative (15.8% [3/19] and 27.9% [12/43] respectively, *P* = 0.311), suggesting that HPV positivity tends to show a low engraftment rate. Drug responses in PDX recapitulated the clinical responses of the matching patients with pan-HER inhibitors and pan-PI3K inhibitor.

**Conclusions:**

Genetically and clinically annotated HNSCC PDXs could be useful preclinical tools for evaluating biomarkers, therapeutic targets, and new drug discovery.

## Background

Head and neck squamous cell carcinoma (HNSCC) is the sixth common malignancy worldwide [1]. Most patients experience local/regional recurrences, and systemic metastases occurred in about 20% of these patients after front-line treatment. Therapeutic options for recurrent or metastatic HNSCC patients include platinum-based combined or single cytotoxic chemotherapy, targeted therapy, and immunotherapy. Despite aggressive treatment regimens, survival outcomes in patients with metastatic disease remains dismal, showing median overall survival of less than one year [1]. Recently, personalized therapy targeting specific genetic alterations has been introduced in HNSCC, and clinical trials are underway [2].

Although preclinical studies presented favorable responses to numerous novel anticancer agents, actual clinical trials in patients have not been as successful, with only 5% of novel anti-cancer agents showing efficacy. These disappointing results emphasize the necessity for novel therapeutic strategies to improve prediction of efficacy on the preclinical stage [3, 4]. Increasing attention has been engrossed on the development and characterization of patient-derived tumor xenograft (PDX) [1]. PDX models are established by direct engraftment of the patient’s tumor into immunodeficient mice. These models recapitulate the principal histologic and genetic properties of corresponding patients’ tumor, and retain more tumor heterogeneity than other preclinical models [5, 6]. Therefore, we can anticipate patient’s clinical outcome by referring to PDXs, and thus are useful for clinical translational research, drug screening, and biomarker discovery and validation. PDXs are in vivo models to test clinically guided hypotheses, capable of evaluating drug activity and novel drug combined strategies, and also to elucidate their predictive biomarkers [7, 8].

p63 is a p53-related DNA-binding protein that helps regulate differentiation and proliferation in epithelial progenitor cells [9]. The expression of p63 is increased in squamous cell cancer of various cancer type including the lung, esophagus, and head and neck but not expressed in adenocarcinoma. In this study, we conducted immunohistochemical staining panel including p16, p63, Ki67 as surrogate marker for diagnosis of squamous cell carcinoma.

Several groups have established PDX models of HNSCC, characterized their genomic fidelity and therapeutic response profiles [10–15]. Herein, we established PDXs using surgically resected or biopsied tumor tissues from HNSCC patients and characterized both histological and genomic fidelities of the established PDXs. We aim to evaluate the clinical factors affecting the establishment of HNSCC PDX models and whether established PDX models can reflect actual patients. Established PDX models in this study faithfully replicated the histologic, genomic, and responses with novel agents (pan-HER inhibitor and pan PI3K inhibitor) observed in the matched HNSCC patients. Our HNSCC PDX repository, which is clinically and genomically annotated in a comprehensive manner, may serve as a genuine platform for testing novel anti-cancer drugs.

## Methods

### Patients

The current research work was approved by the Severance Institutional Review Board (#4–2013-0526), and patients’ tumor tissues were from the Yonsei University Severance Hospital. All patients agreed with written informed consent. We collected tumors and its paired peripheral blood species to establish PDX and further conduct genetic analysis.

### Establishment of PDX models

We established the HNSCC PDX model using a similar method as that used to generate lung cancer PDX [8]. PDX models were generated using 6- to 8-week-old female nude (nu/nu) and severe combined immunodeficient (NOG, NOD/Shi-scid/IL-2Rγ^null^) mice (OrientBio, Seoul, Korea). We carefully removed the necrotic lesion from biopsied specimens with a surgical blade and subcutaneously implanted small pieces (3 × 3 × 3-mm^3^) obtained from each patient into 1–2 mice. When a tumor became a diameter of approximately 1.5 cm, it was dissected and chopped into small specimens (3 × 3× 3-mm^3^) and engrafted into other mice set. We defined the patient-originated specimen as the F0 generation, whereas subsequent generations were numbered consecutively by the number of re-implantations (e.g., F1, F2, or F3) [16]**.** We engrafted patient-derived tumor (F0) into the NOG mice and after F1 generation in nude mice. We usually expanded F3 for drug responses of in vivo PDX models. We assigned Yonsei Human In Mouse (YHIM) identifiers in tumors and related PDXs corresponding to the original patient-derived tumors. After completion of experiments, we sacrificed mice by inhalation of anesthetics with CO_2_. All mice models were maintained in the specific pathogen-free facility of the Avison BioMedical Research Center (ABMRC) Animal Research Center at Yonsei University College of Medicine. We performed all procedures according the Animal Research Committee’s Guidelines at Yonsei University College of Medicine.

### Sample storage

Tumor fragments from original primary tumors and PDX models were sliced into 3 × 3× 3-mm^3^ fragments using a surgical blade in a laminar flow cabinet. For storage, three fragments were placed in RPMI 1640 medium (supplemented with streptomycin, penicillin, gentamicin, and dimethyl sulfoxide), flash frozen, and kept at − 80 °C. The stored fragments were used for genetic profiling and/or conserved in RNAlater RNA stabilization reagent (Qiagen). Moreover, we fixed fragments in 10% neutral-buffered formalin and made paraffin embedded tissue samples for further additional pathological analysis [8].

### Tumor growth measurement

Tumor size was measured once or twice every week in two dimensions with a caliper. Tumor volume was calculated using following formula: (width^2^ × length)/2. When the tumor was measured more than 1 cm^3^ or the mice reached an endpoint written in the Dutch Code of Practice for animal experiments in cancer research [17], the tumor was harvested and stored in media for either preservation or propagation into a following generation. The time of latency was calculated as the duration of time from engraftment until when tumor became measurable (about 70 mm^3^) [8].

### Histology

We fixed harvested tissues from all PDX models in 10% buffered formalin in 30 min of resection. After 24 h of tissue fixation, routine procedures were followed for further tissue processing. Hematoxylin and eosin- and immunohistochemistry (IHC) staining with p63 antibody (Abcam: ab735, clone A4A), Ki67 antibody (Cell Signaling Technology, clone D2H10) stained slides were prepared, and the histopathological characteristics of each tumor including differentiation and tumor necrosis were reviewed pathologist (S.O.Y) in our institution. Moreover, p16 IHC (CINtec Histology, clone E6H4) was performed using a Ventana Bench Mark XT Autostainer to confirm the matched PDX F2 tissue originated from HPV positive patients (YHIM-3001, 3007, 3011). Regarding HPV positivity, we used conventionally accepted criteria and positivity was defined as the presence of strong and diffuse nuclear and cytoplasmic staining in more than 70% of tumor cells [18].

### Targeted deep sequencing

We performed targeted deep sequencing on nine PDX and primary tumor paired specimens.. The SureSelect custom capture probe for the targeted deep sequencing was designed to detect 244 genes which are based on reporting of cancer related journals and literature. The target coverage of the captured region was 1000x, and to accomplish this, we used Illumina HiSeq 2500 platform as paired-end reads. We analyzed the targeted deep sequencing data based on the previous our report [8]. In the general case, PDTX sequences of raw FASTQ were mixed human and mouse genome. For that reason, it is necessary to remove mouse reads from FASTQ. To perform this process, the human(hg19) and mouse(mm10) reference genome were integrated into one reference genome. Then Burrows-Wheeler Aligner [19] was run to identify the best similar read against the human genome refer to the primary alignments. We mapped reads to the most similar regions in both human and mouse genomes and obtained best-matched reads of the human genome [20]. The Genome Analysis Tool Kit ver. 3.5 was used to conduct mark duplication, local realignment, and base quality score recalibration for the obtained reads. Germline mutations were identified using a GATK Haplotype caller. We filtered out variants with poor quality through GATK VariantFiltration and discarded according to the followings: QD < 2.0, DP < 20, FS > 60.0, MQ < 40.0, MQRankSum<− 12.5, ReadPosRankSum<− 8.0, and mutant allele frequency (MAF) < 0.2. Somatic mutations were called by MuTect v.1.1.7 [21] using a default criteria, and databases including CIVIC [22] and DoCM [23]were used to annotate these mutations.

### Oncomine Cancer panel analysis

We used the Oncomine Cancer Panel to identify additional gene fusions and to cross-validate the genetic variations with the PDTX targeted deep sequencing data as described in our previous reports [8, 24]. Fourteen HNSCC and PDX samples (YHIM-3001, YHIM-3014) were sequenced but YHIM-3015 was not because of sample insufficiency. For amplified libraries, the Ion PGM Template kit on a OneTouch v2 was performed using the multiplexed DNA template according to the manufacturer’s protocol. The Torrent Suite Server v4.4 was utilized for the alignment process using the human genome reference(hg19). The variant calling and annotation were performed by self-owned variant analysis system (Ion Reporter Workflow v5.0, Oncomine Panel Annotations set v1.1, Oncomine Panel Baseline v2.0, Oncomine Variant annotator plugin v2.0) which is contained in Torrent Suite Server v4.4. To figure out the pattern of the variants over all sequenced data, we used Oncoprint provided by ComplexHeatmap package [25].

### In vivo drug treatment

As described in previous reports [8], mice were set apart and randomized into each group (six to seven mice per group), when tumor volumes became 200–250 mm^3^. We administered vehicle (5 mM citrate buffer) to the control group. We administered afatinib (15 mg/kg) and BKM120 (35 mg/kg) methotrexate (30 mg/kg) via oral gavage or intraperitoneal injection mixed with 5 mM citrate buffer in deionized water into each model. We measured dimensions of tumor twice weekly using a digital caliper and calculated tumor volume using the following formula: tumor volume = [length×width^2^]/2. We defined percentage tumor growth inhibition as [%TGI = 1 − (change of tumor volume in treatment group/change of tumor volume in control group) × 100] to evaluate antitumor efficacy as primary experimental outcomes.

### Statistical analysis

In vivo drug test, the sample size was six to seven in each group. The data are presented as the mean ± SD. Statistical differences between vehicle and afatinib treatment group were determined using ANOVA test. A *p*-value< 0.05 was considered statistically significant.

## Results

### Establishment of the HNSCC PDX models

We collected 62 tumor specimens from patients and engrafted these specimens into mice between May 2013 and March 2017. Among these, 15 HNSCC PDXs were successfully generated, which include, 8 tongue, 3 oropharyngeal, 1 hypopharyngeal cancers, 1 ethmoid sinus cancer, 1 supraglottic cancer, and 1 parotid gland. Baseline characteristics for the established 15 PDX models are summarized in Supplementary Table 1. Patients were median 55 years old (range: 36–72), and tumor stages ranged from II to IV. Six PDXs (40%) were successfully generated using biopsied tumor tissues from advanced-stage HNSCC patients. Tissue samples were obtained from surgical resection (60%, 9/15) and core biopsy (40%, 6/15). The donor tissue for the 15 established PDX models were derived from primary (6/15, 40%) and metastatic sites (9/15; 60%).

All PDXs were generated through 3–5 sequential transfers with varying latency times from 10 to 41 days. We established a HNSCC PDX repository, consisting of the original patient tissues and the PDX tissues from each transfer, including formalin-fixed paraffin-embedded (FFPE) blocks, viable cryopreserved tissue specimens, and RNAlater-treated and/or fresh-frozen tissues [8].

### PDX models preserve histological stability compared to primary cancers

**Fig. 1:**
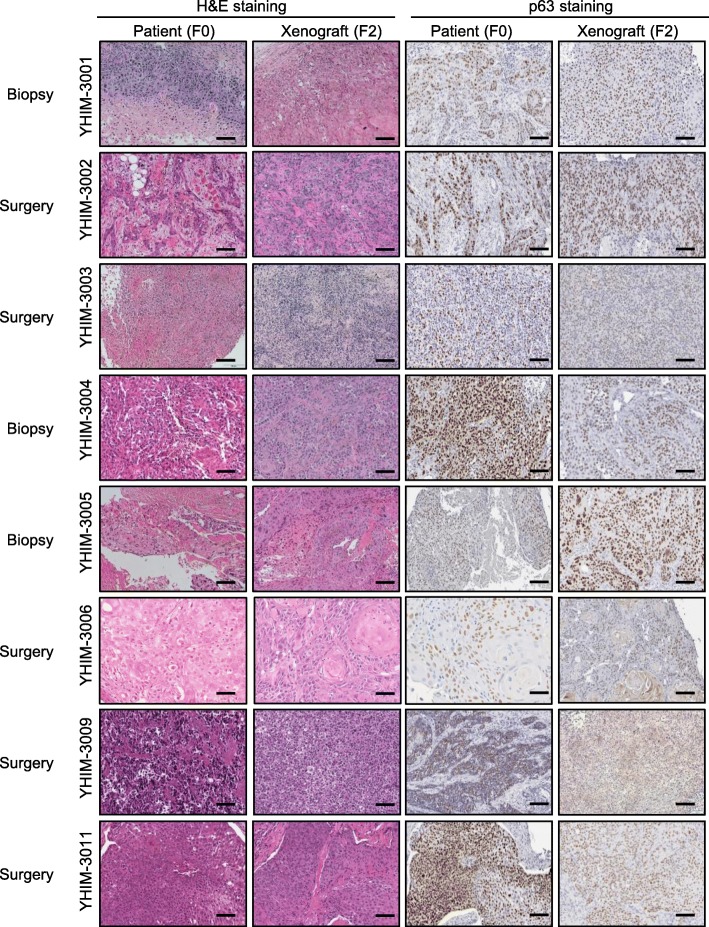
Comparison of histopathologic and immunohistochemistry findings between patients and their matched patient-derived xenografts (PDXs, F2 generation) of head and neck cancer squamous cell carcinoma (HNSCC). Left columns show hematoxylin and eosin-staining and right columns show immunohistochemical staining of p63, a marker of squamous cell carcinoma, from patients and the PDX of each model. Representative stained sections are shown (magnification: 200× in patient samples; scale bars = 100 μm)

In order to determine the biological stability of the PDX tumors, the histological properties from original patient tumors (F0) and matching second-generation PDX tumors (F2) were compared. F0 and F2 generation tumors had close histopathological characteristics including differentiation and tumor necrosis, as well as similar p63 expression (Fig. 1 and Supplementary Figure 1). Detailed pathologic information between F0 and F2 were summarized in Supplementary Table 2. Additionally, we performed the Ki67 IHC staining as surrogate marker of proliferation (Supplementary Figure 2**).** Taken together, the F2 tumors preserved comparable histological characteristics to those of F0 tumor specimens, implicating morphological fidelity with their matched patient tumors. Moreover, we validate HPV positivity in corresponding PDX tumor tissues by IHC staining of p16 protein in tumor cells as shown in Supplementary Figure 3.

### Engraftment and tumor growth rates of HNSCC PDX models

We further analyzed successful engraftment according to tumor acquisition method, tumor acquisition site, stage at tumor acquisition, and human papillomavirus (HPV) infection (Table 1). We defined engraftment success as completion of the transfer to the F3, third generation. We achieved a successful overall engraftment rate of 24% (15/62).

**Table 1:** Univariate analysis to determine the association between covariates and patient-derived xenograft establishment

Variable	PDX establishment		OR (95% CI)	*P-*value
	**No (*n*** **= 47)**	**Yes (*n*** **= 15)**		
**Cancer type**				
Oral cavity cancer (ref)	27 (77.1%)	8 (22.9%)	1.181 (0.367–3.797)	0.780
Others	20 (74.1%)	7 (25.9%)		
**HPV infection**				
P16 negative or unknown (ref)	31 (72.1%)	12 (27.9%)	0.484 (0.119–1.967)	0.311
P16 positive	16 (84.2%)	3 (15.8%)		
**Method of tumor acquisition**^a^				
Biopsy (ref)	0 (0%)	6 (100%)	0.015 (< 0.001–0.148)	0.010
Surgical resection	47 (83.9%)	9 (16.1%)		
**Site of sampling**^a^				
Metastatic (ref)	0 (0%)	9 (100%)	0.007 (< 0.001–0.068)	0.002
Primary	47 (88.7%)	6 (11.3%)		
**Recurrence**^a^				
Primary (ref)	47 (75.8%)	8 (12.9%)	83.842 (3.600–1951.715)	0.006
Recurrent	0 (0%)	7 (11.3%)		
**Stage at tumor acquisition**				
Stage I–III (ref)	24 (85.7%)	4 (14.3%)	2.870 (0.798–10.314)	0.106
Stage IV	23 (67.6%)	11 (32.4%)		

*PDX* patient-derived xenograft, *OR* odds ratio, *HPV* human papilloma virus

^a^ Firth’s method was used for a table with one zero cell count

When analyzing engraftment rates using the acquisition method, biopsy showed a significantly higher engraftment rate than that of surgical resection (100% [6/6] vs. 16.1% [9/56], *P* = 0.010). Establishment using specimens from metastatic sites demonstrated a significantly higher engraftment rate compared to those from primary sites (100% [9/9] vs. 11.3% [6/53], *P* = 0.002). A higher stage (stage IV) at sample acquisition tended to show a higher engraftment rate than that of stage I–III (32.4% [11/34] and 14.3% [4/28], respectively, *P* = 0.106). Of the 62 tumors classified, 19 were HPV-positive, based on p16 protein expression in the major part of cancer cells. We successfully generated three PDX models from HPV-positive tumors, as compared to 12 from HPV-negative tumors (15.8% 3/19 and 27.9% 1243, respectively, *P* = 0.311), suggesting that HPV positivity tends to show a low engraftment rate. Engraftment rates were comparable across primary cancer types.

If we performed the multivariate analysis with several variables including “Method of tumor acquisition”, “Site of sampling”, and “Stage at tumor acquisition”, “Site of sampling” was observed the significant factor related with the success of PDX establishment (OR = 0.016, 95%CI = < 0.001–0.355, *P* = 0.009) (Supplementary Table 3).

**Fig. 2:**
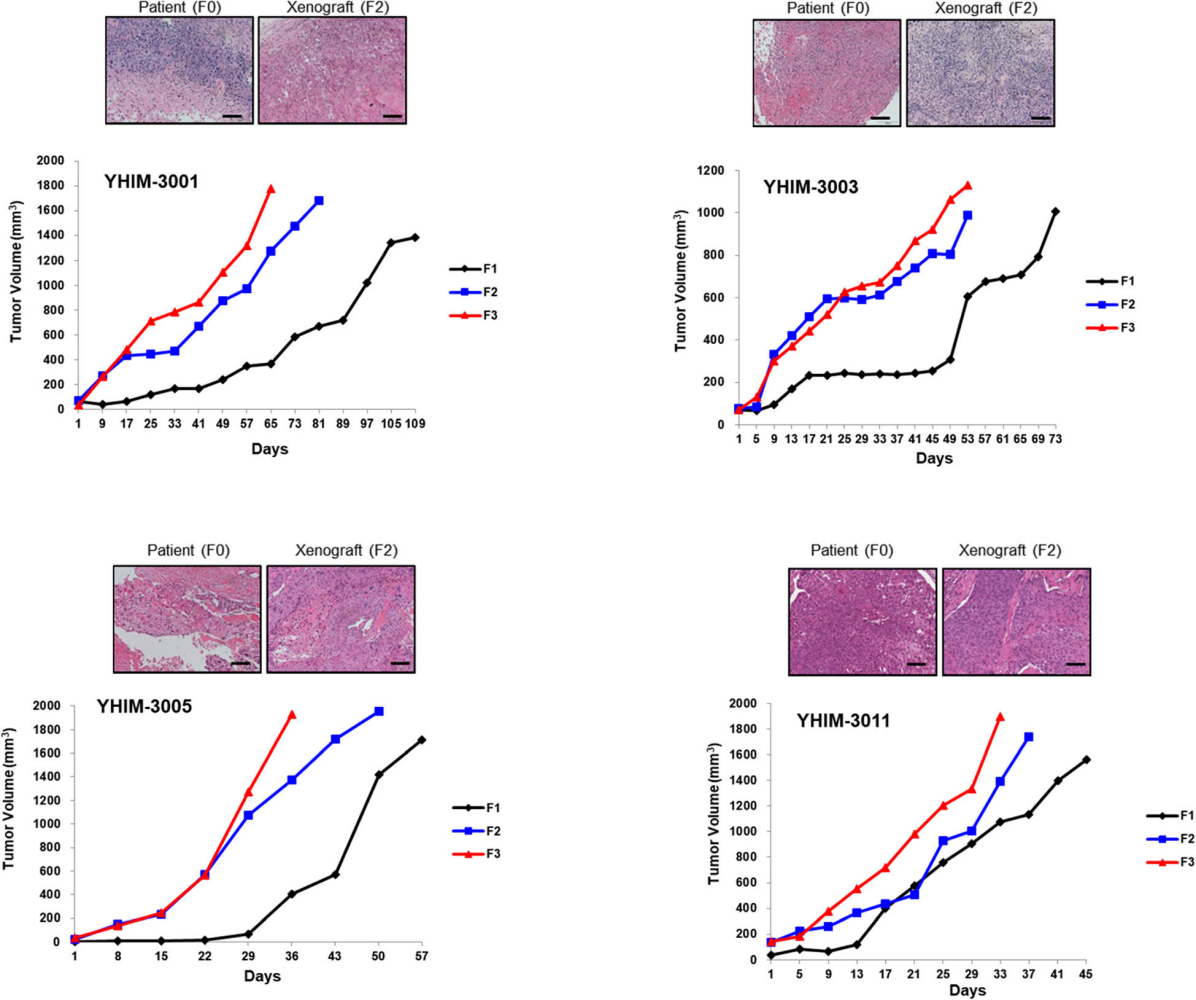
Tumor growth curves of head and neck squamous cell carcinoma (HNSCC) patient-derived xenograft (PDX). PDX tumors were grown in severe combined immunodeficient (NOG) (F1) and nude (nu/nu) mice (F2 and F3). Three passages of xenografts, as represented by F1, F2, and F3, are plotted as tumor volume (mm^3^) over time

Figure 2 illustrates the tumor growth curves in the established HNSCC PDXs for three generations (F1–F3). F3 exhibited relatively faster growth than that of F1 and F2. Moreover, the growth rate stabilized after the third generation. The median latency time was 27 days, but it varied between tumor acquisition method, tumor acquisition site, stage at tumor acquisition, and HPV infection; moreover, samples acquired at stage IV showed a significantly longer latent time than did other sources of variation (Supplementary Table 4).

### Genomic fidelity of the PDX tumors

**Fig. 3:**
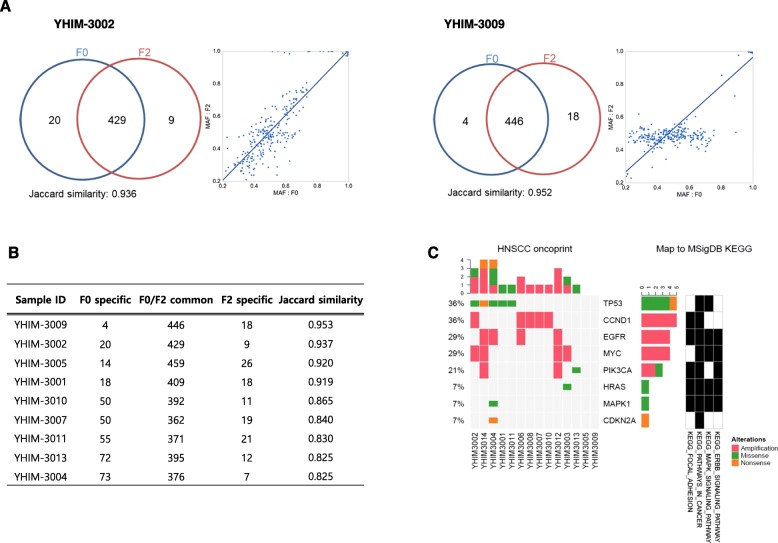
Results of targeted deep sequencing to compare genetic alterations between patient-derived and second-generation tissues (**a**) We used Venn diagrams to demonstrate the overlapping somatic mutations (single-nucleotide variants and insertions/deletions) for YHIM-3002 and YHIM-3009 samples. The Jaccard similarity score was used to measure the similarity; the score of most samples was > 82%. The highest scoring models were YHIM-3002 and -3009, with respective Jaccard similarity scores of 93.6 and 95.2% (Fig. 3**a**). The MAF values for common mutations between F0 and F2 demonstrated overall concordance (*r*^*2*^ = 0.93 and 0.95 for YHIM-3002 and -3009, respectively), implicating that most germline mutations in the F2 sample corresponded with those in the F0 sample (Fig. 3**b**). Heatmaps showing mutational overview of known functionally active genes and significantly mutated genes in patient-derived xenografts (PDXs). Oncomine Cancer Panel was used to detect somatic mutations in *PIK3CA*, *HRAS*, and *TP53*, as well as *EGFR*, *CCND1*, *MYC*, and *PIK3CA* amplification. Heat maps showed all Oncomine-defined relevant alterations in the RNA (header) and DNA components of the 14 PDX specimens. Red indicates amplification, green indicates a missense mutation, orange indicates a small insertion/deletion, purple indicates a fusion, and black indicates a multi-hit result (Fig. 3**c**)

We conducted targeted next-generation sequencing to figure out single-nucleotide variants and small insertions/deletions using nine pairs of the original patient (F0) and the PDX F2 tumor. The goal of current study was to discover identical mutations in F0 and F2, which would indicate that F0 and F2 are identical if most of the germline mutations coincided. The Jaccard similarity score was used to measure the similarity; the score of most samples was > 82%. The highest scoring models were YHIM-3002 and -3009, with respective Jaccard similarity scores of 93.6 and 95.2% (Fig. 3a). The MAF values for common mutations between F0 and F2 demonstrated overall concordance (*r*^*2*^ = 0.93 and 0.95 for YHIM-3002 and -3009, respectively), implicating that most germline mutations in the F2 sample corresponded with those in the F0 sample (Fig. 3b). *HRAS* G12D and *PIK3CA* H1047R mutations were observed simultaneously in F0 and F2 (YHIM-3003, − 3013). F0 and F2 showed similar copy numbers for *CCND1* amplification.

### Genetic alteration based on Oncomine Cancer panel

We next performed a genomic overview of the most recurring somatic mutations of interest in 14 PDX tumor specimens using the Oncomine Cancer Panel (Fig. 3c, Supplementary Table 5). After filtering the predefined Oncomine variants, we found an average of 0.64 relevant somatic point mutations and 1.1 high-level CNAs per specimen. An integrative heat map showed that the prioritized alterations across the YHIM cohort and the copy number profiles for all samples (Fig. 3c). All Oncomine-derived relevant genetic alterations in the RNA and DNA components of the 14 PDX specimens are shown in the heat map. *PIK3CA*, *HRAS*, and *TP53* mutations and *EGFR*, *CCND1*, *MYC*, and *PIK3CA* amplifications were identified in established PDXs. Gene amplifications included *CCND1* (36% [5/14]), *EGFR* (29% [4/14]), *MYC* (29% [4/14]), and *PIK3CA* (14% [2/14]). Regarding somatic mutations, *TP53* mutation (36% [5/14], H193R, I195T, R248W, R273H, E298X), *PIK3CA* mutation (7% [1/14], 1047R), and *HRAS* mutation (7% [1/14], G12D) were observed.

### PDXs faithfully recapitulated the anti-cancer efficacy of their matched patients

**Fig. 4:**
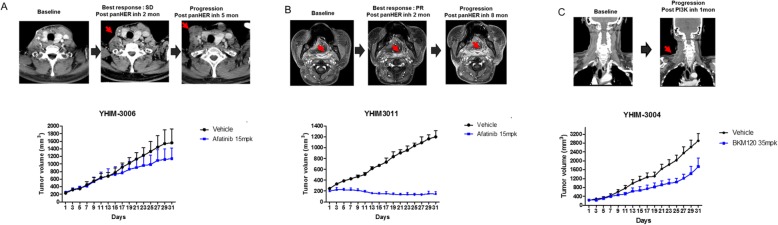
Drug response shown in three patient-derived xenograft (PDX) models. **a** YHIM-3006 was established from a tongue cancer patient who was treated with pan-HER inhibitor and showed stable disease for 5 months. Afatinib treatment produced substantial tumor regression in YHIM-3006, which was concordant with the clinical response in the matched patient. **b** YHIM-3011 was treated with pan-HER inhibitor and showed significant tumor regression, which recapitulated the drug response of the corresponding patient who showed a partial response for more than 8 months. **c** YHIM-3004 was treated with pan-PI3K inhibitor and represented abrupt increase of multiple lymphnode, that mimicked the treatment response of the matched patient showing a progression after 1 month of BKM120 therapy

We then assessed whether the generated PDXs can recapitulate the anti-cancer efficacy of matched patients and thus work for a genuine platform for novel anti-cancer drug efficacy testing. We had two PDX models (YHIM-3006, and YHIM-3011) which were involved in co-clinical trial with afatinib, a pan-HER inhibitor. YHIM-3006 was established using surgical resection from tongue cancer patients. In YHIM-3006, afatinib induce growth delay (TGI = 44.9%; *P* < 0.001) (Fig. 4a), which was similar with the modest clinical outcome in the actual corresponding patient. This patient showed stable disease as best response for 5 months, but the tumor was abruptly increased after 5 months of afatinib treatment. Next, YHIM-3011 was generated using small biopsied tissue from a patient with recurrent oropharyngeal cancer after surgery and radiation therapy and platinum-based chemotherapy. Corresponding patient of YHIM-3011 represented marked tumor shrinkage showing partial response for 8 months, which was recapitulated with the PDX model which showed remarkable tumor shrinkage with afatinib treatment (TGI = 105.7%; P < 0.001) (Fig. 4b). Moreover, these two patients were previously treated with MTX as anti-cancer therapy and showed strong resistance to MTX (Supplementary Figure 4). These findings were correlated with those of MTX response in the matched PDX models. Moreover, YHIM-3004, PDX model were treated with BKM120 as pan-PI3K inhibitor as co-clinical trial. This model showed strong resistance to BKM120 monotherapy, which is recapitulated with response of corresponding patient. This patient showed rapid progression after 1 month to BKM120 monotherapy showing abrupt increase of multiple metastatic LN (Fig. 4 c). The weights of mice in each group were not significantly reduced (data not shown).

We need to interpret cautiously due to small sample size, PDXs seem to recapitulate the drug responses of the corresponding patients and may serve as a clinically relevant platform for novel drug testing.

## Discussion

The present study successfully established 15 HNSCC PDXs by directly engrafting tumor tissue samples from patients into immunodeficient mice. These PDXs maintained features of the donor patients’ cancers as determined by histology as well as genetic aberrations. These established PDXs may be useful as preclinical platforms to test newly developed anti-cancer drug efficacy. Of the 15 established PDXs, most were derived from tongue cancer; six were made using a small amount of biopsied tissue originated from patients with advanced HNSCC. The established PDXs harbored some clinically implicated druggable targets, including copy number amplifications (*CCND1*, *PIK3CA, EGFR*) and somatic mutations (*PIK3CA* H1047R, *HRAS* G12D).

Our data suggest that HNSCC PDX establishment has a 24% (15/62) success rate. We comprehensively evaluated which factors, including tumor acquisition method, tumor acquisition site, stage, and HPV infection, affected the engraftment rate of PDX models. PDXs from a biopsy sample, from a metastatic site, or of a higher stage showed a significantly higher engraftment rate. Additionally, PDXs from HPV-negative tumors tended to show a higher engraftment rate compared with those from HPV-positive tumors. These results of our study are consistent with prior reports [13].

We conducted targeted next-generation sequencing to figure out single-nucleotide variants and small insertions/deletions using nine pairs of the F0 and the PDX F2 tumor. We observed that genetic mutations or amplifications of PDX matched to those of original tumors. However, it could be possible that tumors with stable mutation/amplification were selected during the course of PDX establishment. To find out the identification of PDX models and their matched patients, we exam histology and IHC staining using p63 and p16 in this study. Other reports also evaluate the histology and IHC staining including p16 and CD31, to identify whether established PDX models retain the histopathologic characteristics of original patients [26, 27].

The essential strength of the PDX platform is that it is generated from unselected and uncultured patient samples and has the homogeneous genetic background as its matched patient tumor [28]. In our study, 83–95% of genetic aberrations and the morphology from the corresponding donor patient cancers were maintained in the established PDXs. This suggests that PDXs maintain relatively stable genomes without significant accumulation of DNA structure rearrangements, but do have some of enrichment for PDX-specific single nucleotide variants [29]. Therefore, we conclude that anti-cancer efficacy research works performed using PDXs will be similar to what actually happens in patient tumors. Previous studies have also shown great concordance of response between donor patient tumors and PDXs [30, 31]. The concordance between patient and xenograft responses despite the absence of the immune system is encouraging and consistent with previous literature on targeted therapy [10, 11]. In this study, drug responses in PDX recapitulated the clinical responses of the corresponding patients with afatinib. Since clinical studies are examining the combination of Afatinib with RT, future studies examining the combination in their PDX panel would be relevant. In this regard, previous studies have shown concordance of radiation response of HPV+ and HPV- PDX models of HNSCC [14]. In this study, the responses of PDXs to pan-HER inhibitor mimicked the clinical responses of the matched patients, indicating that established PDXs represent a clinically relevant platform for predicting drug response.

HPV infection has become as a major risk factor, specifically in the oropharynx, accounting for more than 20% of all HNSCCs [32]. While the overall HNSCC incidence continues to decline, there has been an observable and highly increase in the incidence of HPV-associated HNSCC, predominantly among young patients [33–35]**.** Of the 62 tumors classified, 19 were HPV-positive. We successfully established three PDX models (15.8% [3/19] engraftment rate) from HPV-positive tumors, suggesting that HPV positivity decreases the engraftment rate (HPV-negative tumors: 27.9%, [12/43]). Our HPV-positive PDXs could help identify novel and more effective therapeutic strategies and transition patients with these tumors to personalized therapies. Interestingly, YHIM-3001 and YHIM-3011 models were originated from HPV positive and never smoker tongue and oropharyngeal cancer patients. Additionally, these two models have I195T and H193R mutation in TP53 gene, which is unusual. These models (YHIM-3001 and 3007) were originated from patients with recurrence who were previously treated with chemotherapy. Therefore, the presence of TP53 mutation could be explained to be chemotherapeutic agents induced mutations.

The Cancer Genome Atlas (TCGA) with comprehensive genetic profiling of 279 HNSCC specimens demonstrated that *EGFR*, *CCND1*, *PIK3CA*, and *FGFR1* amplifications and *TP53*, *CDKN2A*, and *HRAS* mutations are common genetic alterations in HNSCC [36]. These genetic alterations are potential therapeutic and druggable targets in HNSCC patients. EGFR as a cell surface receptor member of the ErbB family has been extensively studied in HNSCC. Genetic alterations in EGFR pathway are potential therapeutic and druggable targets in HNSCC. Currently, EGFR targeting strategies include EGFR antibody (i.e., cetuximab) and EGFR tyrosine kinase inhibitors including erlotinib, gefitinib, or afatinib [37]. Although several prospective clinical trials were conducted, only a minor portion of R/M HNSCC responded to EGFR targeting agents; predictive molecular biomarkers have not yet been identified [37]. Furthermore, genetic alterations in the PI3K signaling pathway are commonly observed in HNSCC, and pan-PI3K inhibitor such as buparlisib with paclitaxel improved efficacy in R/M HNSCC patients compared with paclitaxel alone, suggesting that PI3K inhibition plays an important anti-cancer role. However, predictive markers to determine the clinical efficacy for PI3K inhibitors are unknown [38]. To maximize the treatment outcome of targeted therapy in HNSCC patients, there is a need to identify molecular determinants for drug efficacy. In this study, clinically relevant therapeutic targets such as *CCND1*, *PIK3CA*, and *EGFR* amplifications and *PIK3CA* H1047R, *HRAS* G12D mutations were observed in several PDX models. These PDX models that retain the potential therapeutic target can help to guide efficient targeted therapy and overcome resistance mutations. Recently, several umbrella clinical trials, in which patients were allocated to targeted therapy according to genetic alterations based on next-generation sequencing, are underway for advanced HNSCC. The MOSCATO-01 clinical trial demonstrated that genetic analyses of advanced solid cancers patients improved treatment outcomes through matched targeted therapeutics [39]. The ongoing NCI-MATCH trial and TRIUMPH trial (NCT03292250) are evaluating whether molecular biomarkers can predict response to target therapy in advanced cancer patients [24, 40]. This process might render greater success in identifying more potent treatment strategies and transitioning patients to personalized therapies.

Notably, the PDX models have several advantages in precision medicine in translational cancer research. Conventional cell lines are usually cultured in vitro for a long time, which leads to the development of extra genetic alterations that differ from the original cancer. Thus, it is difficult to predict clinical anti-cancer efficacy only by relying on the genomic information of cell lines (i.e., the Genomics of Drug Sensitivity in Cancer [41] and the Cancer Cell Line Encyclopedia [42] [43]. Moreover, PDXs may aid in identifying drug–tumor relationships on the basis of inter- or intra-patient tumoral heterogeneity. Prior report showed that intertumoral angiogenic heterogeneity was observed in HNSCC PDX models and the therapeutic response anti-VEGF therapy was dependent on their angiogenic heterogeneity [44].

In addition, it is feasible to predict and overcome acquired resistance to novel anti-cancer drugs by investigating resistance mechanisms. Thus, investigators can synchronize preclinical experiments and clinical trials and systematize study design and data analysis protocols for PDX [8]. Recent development of high-throughput technologies provided important molecular insights in genetically characterized PDX models.

On the other hand, there are some disadvantages of the PDX models [45]. First, it routinely takes 4–5 months to generate and stabilize the first generation of PDX (F1), but only 2–5 weeks for subsequent passages. This long time lapse means that PDX platform can only be used as a research tool, especially for cancer patients with a fast disease progression and a short survival time [46–48]. Second, PDX platforms lack an intact immune system and may be of limited use for immunotherapeutic drug testing. These limitations could be solved by establishing humanized in vivo models, that were used to evaluate anti-cancer drug efficacy and elucidate predictive biomarkers and resistant mechanisms to immunotherapies.

## Conclusion

In summary, we have developed and characterized an HNSCC PDX models that is useful for screening novel targeted drugs or combination drugs and identifying resistance mechanisms. Our data illustrate that PDXs may be a robust preclinical platform for analyzing biomarkers, therapeutic targets, and novel anti-cancer drug development.

## Supplementary information

**Additional file 1 **: **Supplementary Table 1**. Baseline characteristics of patients (N = 15). **Supplementary Table 2**. Detailed pathologic information of F0 and F2 generation. **Supplementary Table 3.** Multivariate analysis to determine the association between covariates and patients- derived xenograft establishment. **Supplementary Table 4.** Latency time of 15 established PDX models. **Supplementary Table 5.** Detailed information of established head and neck cancer patient-derived xenografts

**Additional file 2 **: **Supplementary Figure 1.** Comparison of histopathologic and immunohistochemistry findings between patients and their matched patient-derived xenografts (PDXs, F2 generation) of head and neck cancer squamous cell carcinoma (HNSCC). Left columns show hematoxylin and eosin-staining and right columns show immunohistochemical staining of p63, a marker of squamous cell carcinoma, from patients and the PDX of each model. Representative stained sections are shown (magnification: 200× in patient samples; scale bars = 100 μm).**Supplementary Figure 2.** P16 immunohistochemistry staining of tumor tissue between patient and PDX model. **Supplementary Figure 3.** Ki67 immunohistochemistry staining on tumor tissue of patient derived xenograft model. **Supplementary Figure 4.** Tumor growth rate to anti-cancer therapy in YHIM-3006 and 3011 models.

## Data Availability

The datasets used and/or analyzed during the current study available from the corresponding author on reasonable request.

## Abbreviations

AAALAC: Association of Assessment and Accreditation of Laboratory Animal Care
HNSCC: Head and neck squamous cell carcinoma
HPV: Human papillomavirus
PDX: Patient-derived tumor xenograft
