# Supplementary material for: Establishment and characterization of patient-derived xenografts as paraclinical models for head and neck cancer
Source: BMC Cancer. 2020 Apr 15;20:316. doi: 10.1186/s12885-020-06786-5 (PMC7160896; doi:10.1186/s12885-020-06786-5)
Supplement: Supplementary file 1 — Additional file 1 : Supplementary Table 1. Baseline characteristics of patients (N = 15). Supplementary Table 2. Detailed pathologic information of F0 and F2 generation. Supplementary Table 3. Multivariate analysis to determine the association between covariates and patients- derived xenograft establishment. Supplementary Table 4. Latency time of 15 established PDX models. Supplementary Table 5. Detailed information of established head and neck cancer patient-derived xenografts [file 12885_2020_6786_MOESM1_ESM.docx]

| **Supplementary Table 1. Baseline characteristics of patients (N = 15)** | |
| --- | --- |
| Characteristics | Value (%) |
| Age (years) |  |
| Median | 55 |
| Range | 36-72 |
| Sex |  |
| Female | 5 (33.3%) |
| Male | 10 (66.7%) |
| Primary cancer |  |
| Oral cavity | 8 (53.3%) |
| Oropharynx | 3 (20%) |
| Others* | 4 (26.7%) |
| Smoking history |  |
| Current | 7 (46.6%) |
| Ex-smoker | 3 (20%) |
| Never smoker | 5 (33.4%) |
| Smoking dose |  |
| Median (pack years) | 10 (0–54) |
| Stage |  |
| Stage I–III | 4 (26.6%) |
| Stage IV | 11 (73.4%) |
| Method of tumor acquisition |  |
| Surgical resection | 9 (60%) |
| Biopsy | 6 (40%) |
| Site of sampling |  |
| Primary | 5 (33.3%) |
| Metastatic | 10 (66.7%) |
| *includes hypopharynx (n=1), ethmoid sinus (n=1), parotid gland cancer (n=1), glottic cancer (n=1). | |

| **Supplementary Table 2. Detailed pathologic information of F0 and F2 generation.** | | | | | | | |
| --- | --- | --- | --- | --- | --- | --- | --- |
|  | F0 | | | F2 | | | |
|  | Histology/ Differentiation | Tumor necrosis^a^ | P63^a^ | Histology/ Differentiation | Tumor necrosis^a^ | P63 ^b^ | Ki67^c^ |
| YHIM-3001 | keratinizing moderately differentiated squamous cell carcinoma | 40~50% | 30~40% | keratinizing moderately differentiated squamous cell carcinoma | no | 70~80% | 30~40% |
| YHIM-3002 | keratinizing poorly differentiated squamous cell carcinoma | no | 50~60% | keratinizing poorly differentiated squamous cell carcinoma | no | 80~90% | <10% |
| YHIM-3003 | non-keratinizing poorly differentiated squamous cell carcinoma | 80~90% | 30~40% | non-keratinizing poorly differentiated squamous cell carcinoma | 50% | 50~60% | 20~30% |
| YHIM-3004 | keratinizing poorly differentiated squamous cell carcinoma | no | >90% | keratinizing poorly differentiated squamous cell carcinoma | no | 60~70% | 10~20% |
| YHIM-3005 | keratinizing poorly differentiated squamous cell carcinoma | 20~30% | 60~70% | keratinizing poorly differentiated squamous cell carcinoma | 20~30% | >90% | 30~40% |
| YHIM-3006 | keratinizing moderately differentiated squamous cell carcinoma | no | 30-40% | keratinizing moderately differentiated squamous cell carcinoma | no | 30~40% | 10~20% |
| YHIM-3007 | keratinizing well differentiated squamous cell carcinoma | no | 20~30% | keratinizing well differentiated Squamous cell carcinoma | 90~95% | 20-30% | NA^d^ |
| YHIM-3008 | keratinizing moderately differentiated Squamous cell carcinoma | 10~20% | 80~90% | keratinizing moderately differentiated Squamous cell carcinoma | 90% | 80~90% | 10~20% |
| YHIM-3009 | non-keratinizing poorly differentiated squamous cell carcinoma | 10~20% | 30~40% | non-keratinizing poorly differentiated squamous cell carcinoma | no | <10% | 50~60% |
| YHIM-3010 | keratinizing moderately differentiated Squamous cell carcinoma | no | >90% | keratinizing moderately differentiated Squamous cell carcinoma | 70%. | >90% | 10~20% |
| YHIM-3011 | keratinizing moderately differentiated squamous cell carcinoma | no | 80~90% | keratinizing moderately differentiated Squamous cell carcinoma | no | 80~90% | 20~30% |
| YHIM-3012 | keratinizing poorly differentiated squamous cell carcinoma | no | <10% | keratinizing poorly differentiated Squamous cell carcinoma | no | <10% | 30~40% |

^a^The percentage is semiquantitatively scored for tumor nuclei which express p63 among the entire viable tumor nuclei. Necrotic tumor area is not scored.

^b^ The percentage is semiquantitatively scored for tumor nuclei which express Ki67(MIB1) among the entire viable tumor nuclei. Necrotic tumor area is not scored.

^c^ In PDX of 3007, the Ki-67 scoring was not applicable due to near total necrosis of tumor.

^d^ Tumor necrosis was semiquantitatively measured regarding tumor necrosis over entire tumor areas in a case. To compare the tumor histology of patient and PDX, however, representative images were captured in areas where viable tumor cells are shown.

**Supplementary Table 3. Multivariate analysis to determine the association between covariates and patients- derived xenograft establishment.**

| **Variable** | **OR (95% CI)** | ***P-*value** |
| --- | --- | --- |
| **Method of tumor acquisition** * |  |  |
| Biopsy (ref) | 0.054 (<0.001 - 3.977) | 0.184 |
| Surgical resection |  |  |
| **Site of sampling** * |  |  |
| Metastatic (ref) | 0.016 (<0.001 - 0.355) | **0.009** |
| Primary |  |  |
| **Stage at tumor acquisition** |  |  |
| Stage I–III (ref) | 1.437 (0.267-7.722) | 0.672 |
| Stage IV |  |  |

OR, odds ratio

* Firth’s method was used for a table with one zero cell count.

**Supplementary Table 4. Latency time of 15 established PDX models**

| Variable | Latency time  Median days (range) | *P* value |
| --- | --- | --- |
| Cancer type |  |  |
| Oral cavity cancer | 21 (7–48) | 0.332 |
| Oropharyngeal cancer | 23 (13–28) |  |
| Others | 34 (21–50) |  |
| HPV infection |  |  |
| P16 positive | 24 (13–41) | 0.888 |
| P16 negative/unknown | 25 (7–50) |  |
| Method of tumor acquisition |  |  |
| Surgical resection | 24 (7–50) | 0.869 |
| Biopsy | 26 (13–41) |  |
| Site of sampling |  |  |
| Primary | 26 (10–50) | 0.897 |
| Metastatic | 24 (7–41) |  |
| Stage at tumor acquisition |  |  |
| Stage I–III | 11 (7–17) | 0.0142 |
| Stage IV | 30 (13–50) |  |

HPV, human papilloma virus.

**Supplementary Table 5. Detailed information of established head and neck cancer patient-derived xenografts**

| **Case No.** | **Histology** | **Primary** | **Smoking history** | **HPV (p16)** | **Stage at tumor acquisition** | **Tumor acquisition method** | **Tumor acquisition site** | **Mutation type** | **Prior therapy** |
| --- | --- | --- | --- | --- | --- | --- | --- | --- | --- |
| YHIM-3001 | SQ | Tongue | Never | Positive | Stage IV | Biopsy | Paraspinal muscle | TP53 (I195T) | Surgery, RT, 5FU/DDP |
| YHIM-3002 | SQ | Tongue | Never | Negative | Stage III | Surgery | Tongue | TP53 (R248W), MYC amp, CCND1 amp | Surgery |
| YHIM-3003 | SQ | Tongue | Never | Negative | Stage IVA | Surgery | Tongue | HRAS (G12D), MYC amp | Surgery |
| YHIM-3004 | SQ | Hypopharynx | Never | Negative | Stage IVB | Biopsy | Tongue | TP53 (R273H) | Induction Docetaxel/DDP  Surgery, CCRT |
| YHIM-3005 | SQ | Ethmoid sinus | Current | Negative | Stage IVB | Biopsy | Skin nodule | WT | CCRT, 5FU/DDP |
| YHIM-3006 | SQ | Tongue | Current | Negative | Stage III | Surgery | Lymph node | CCND1 amp, EGFR amp | Surgery, CCRT |
| YHIM-3007 | SQ | Tongue | Current | Positive | Stage II | Surgery | Lymph node | CCND1 amp | Surgery, CCRT |
| YHIM-3008 | SQ | Tongue | Ex | Negative | Stage III | Surgery | Tongue | CCND1 amp | Surgery, |
| YHIM-3009 | SQ | Supraglottic | Current | Negative | Stage IVA | Surgery | Larynx | WT | Surgery, |
| YHIM-3010 | SQ | Tongue | Ex | Negative | Stage IVA | Surgery | Tongue | CCND1 amp | Surgery, |
| YHIM-3011 | SQ | Oropharynx | Never | Positive | Stage IVA | Biopsy | Skin nodule | TP53 (H193R) | Surgery, CCRT, 5FU/DDP |
| YHIM-3012 | SQ | Oropharynx | Ex | Negative | Stage IV | Biopsy | Lymph node | PIK3CA amp, EGFR amp, MYC amp | Surgery, CCRT, 5FU/DDP |
| YHIM-3013 | SQ | Parotid gland | Current | Negative | Stage IV | Surgery | External auditory canal | PIK3CA (H1047R) | Surgery, CCRT |
| YHIM-3014 | SQ | Tongue | Current | Negative | Stage IVA | Biopsy | Chest wall | PIK3CA amp, EGFR amp, MYC amp, TP53 ( E298X) | Surgery, CCRT, 5FU/DDP |
| YHIM-3015 | SQ | Oropharynx | Current | Negative | Stage IV | Surgery | Lymph node | ND | Surgery, CCRT |

HPV, human papilloma virus; SQ, squamous; WT, wild-type; RT, radiation therapy; 5FU,5 fluorouracil; DDP, cisplatin; CCRT, concurrent chemoradiation; ND, not done
