# Supplementary material for: Establishment and characterization of patient-derived xenografts as paraclinical models for head and neck cancer
Source: BMC Cancer. 2020 Apr 15;20:316. doi: 10.1186/s12885-020-06786-5 (PMC7160896; doi:10.1186/s12885-020-06786-5)
Supplement: Supplementary file 2 — Additional file 2 : Supplementary Figure 1. Comparison of histopathologic and immunohistochemistry findings between patients and their matched patient-derived xenografts (PDXs, F2 generation) of head and neck cancer squamous cell carcinoma (HNSCC). Left columns show hematoxylin and eosin-staining and right columns show immunohistochemical staining of p63, a marker of squamous cell carcinoma, from patients and the PDX of each model. Representative stained sections are shown (magnification: 200× in patient samples; scale bars = 100 μm).Supplementary Figure 2. P16 immunohistochemistry staining of tumor tissue between patient and PDX model. Supplementary Figure 3. Ki67 immunohistochemistry staining on tumor tissue of patient derived xenograft model. Supplementary Figure 4. Tumor growth rate to anti-cancer therapy in YHIM-3006 and 3011 models. [file 12885_2020_6786_MOESM2_ESM.docx]

**Supplementary Fig. 1.**


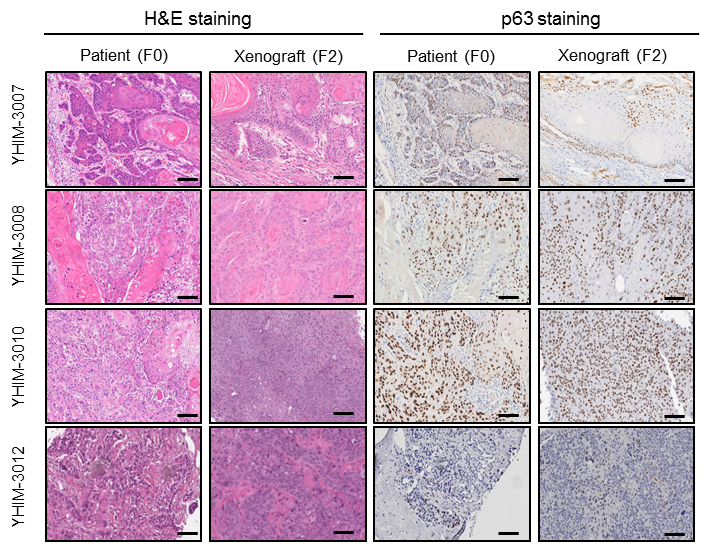


Comparison of histopathologic and immunohistochemistry findings between patients and their matched patient-derived xenografts (PDXs, F2 generation) of head and neck cancer squamous cell carcinoma (HNSCC). Left columns show hematoxylin and eosin-staining and right columns show immunohistochemical staining of p63, a marker of squamous cell carcinoma, from patients and the PDX of each model. Representative stained sections are shown (magnification: 200× in patient samples; scale bars = 100 µm).

**Supplementary Fig. 2.**


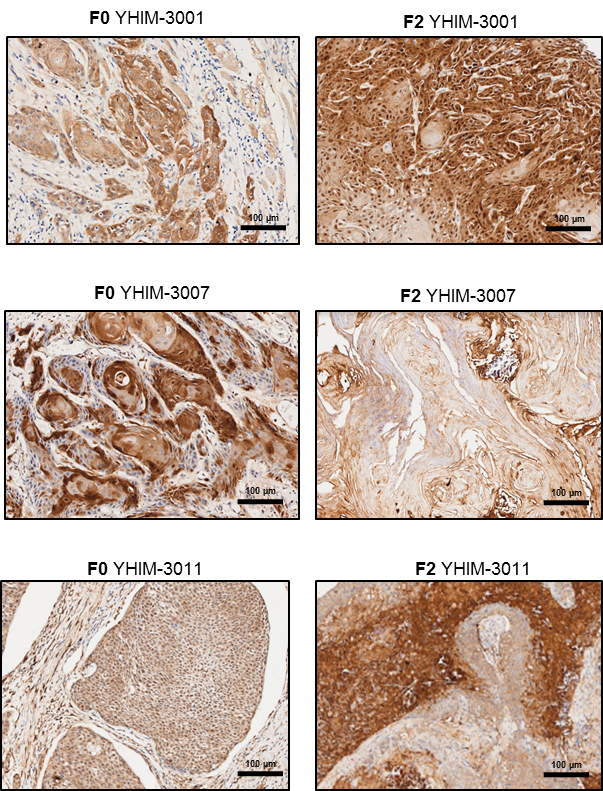


P16 immunohistochemistry (IHC) staining of tumor tissue between patient and PDX model. Left columns show P16 IHC of F0 and right columns show P16 IHC of F2.

**Supplementary Fig. 3.**


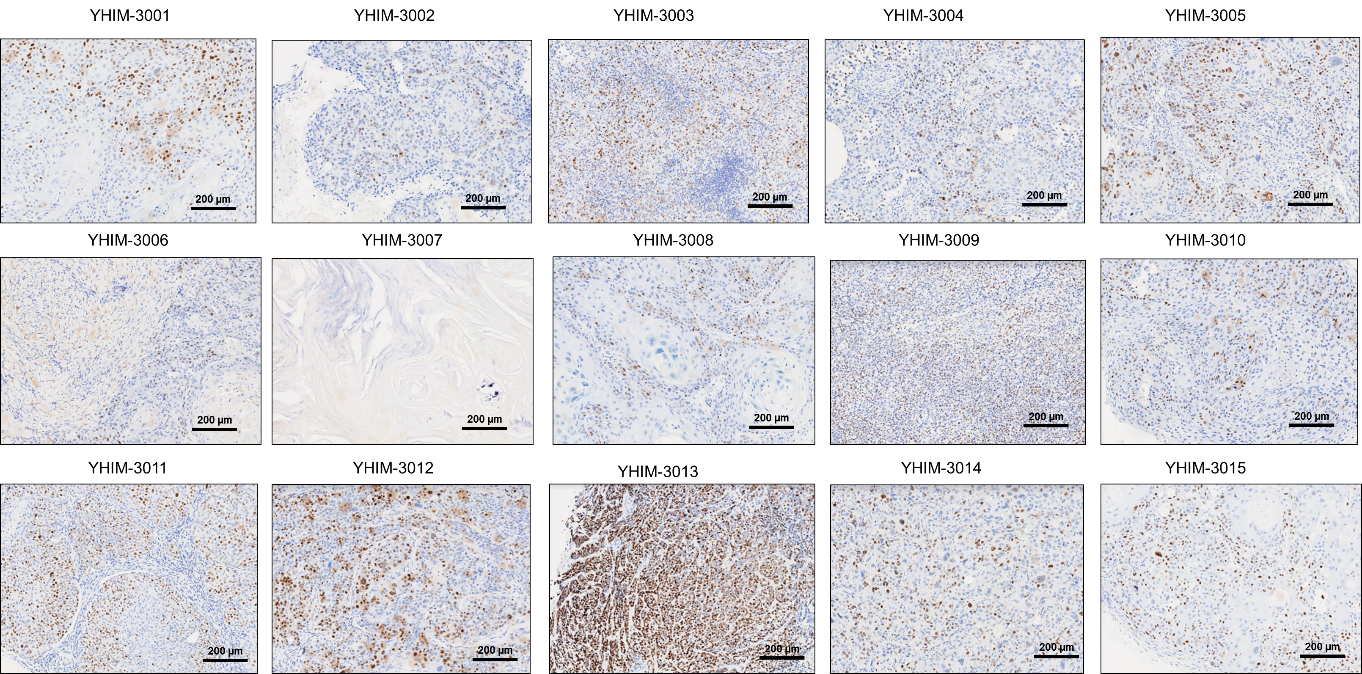


Ki67 immunohistochemistry staining on tumor tissue of patient derived xenograft model.

**Supplementary Fig. 4.**


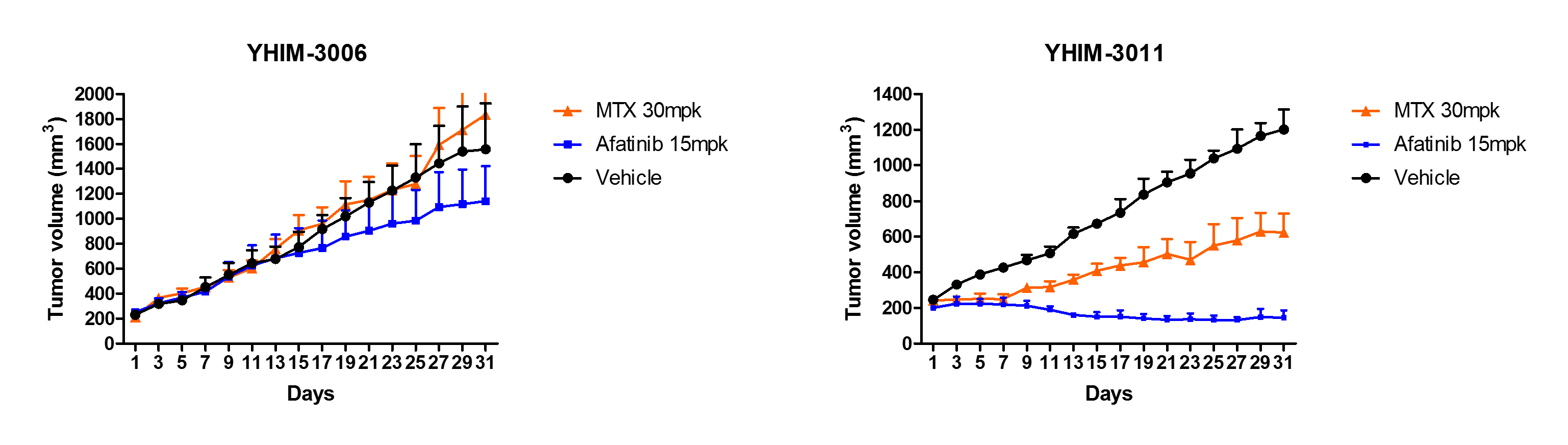


Tumor growth rate to anti-cancer therapy in YHIM-3006 and 3011 models.
